# Transgender Healthcare and Medical Education: An Inductive Thematic Analysis of Digital Discourse

**DOI:** 10.7759/cureus.34972

**Published:** 2023-02-14

**Authors:** Aakash Reddy

**Affiliations:** 1 College of Natural Sciences, University of Texas at Austin, Austin, USA

**Keywords:** inductive thematic analysis, transgender and gender-diverse, health disparities, medical education, transgender health

## Abstract

Introduction

Transgender and gender-diverse (TGD) patients face significant healthcare disparities informed by discrimination, social stigma, and socioeconomic inequality. This is further exacerbated by a paucity of transgender health content in medical education, contributing to a large proportion of providers being ill-equipped to manage TGD patients’ health concerns.

Method

This paper uses the framework approach for an inductive thematic analysis of online discussion forums concerning transgender health and medical education. Online text from anonymous participants was chosen for their authentic accounts of healthcare experiences and to ensure responses are less restrained in discussing sensitive topics.

Results

Three major themes were identified from the data: desire for more knowledgeable providers, negative healthcare experiences and attitudes, and recommendations for transgender health education. Three subcategories emerged from the latter theme that further illuminated participant recommendations for inclusive healthcare.

Conclusions

As a result of the unique healthcare concerns faced by TGD patients, providing materials to educate students and providers is crucial to meet the needs of the TGD patient population. Participant reports align with previous literature in recommending curricular reforms in medical school, promoting career options for TGD-identifying people in healthcare, and cultivating a more inclusive clinical culture.

## Introduction

Transgender health

Transgender and gender-diverse (TGD) patients face significant barriers to equitable healthcare [1]. Transgender patients express a different gender identity from their birth-assigned sex, and gender-diverse is an umbrella term to characterize those who have a gender identity and expression that does not match normative expectations of a gender role [1]. Although estimates can vary based on the definition of transgender, up to 35 million people identify as transgender on a global scale [1]. In the United States alone, approximately 1.4 million people identify as transgender, which has nearly doubled from prior estimates in 2011 [2].

One major contributing factor to this surge in TGD self-identification is the online discourse on gender identity [3,4]. Participants join forums and support groups and involve themselves in discursive identity development with other community members. One study found that more TGD participants used online forums than in-person support groups, with over 98% of its participants naming online media as important sources of gender identity information. Participants reported feeling more satisfied with their identity after involving themselves in online forums [5]. As informed by discussion spaces in online forums, a clear trend reveals a growing number of people openly identify as TGD.

However, discriminatory practices, social stigma from family and peers, and socioeconomic inequality promote significant healthcare disparities for TGD patients. In a survey in the United States (N = 2,100), transgender people were found to have significantly lower employment rates, lower incomes, and higher poverty rates when compared to cisgender men [6]. In another national survey of TGD people in the United States (N = 27,715), a third of respondents (29%) reported living in poverty, nearly three times the rate of the general US population (12%). A similar proportion of respondents also reported experiencing homelessness (30%), with 12% reporting being homeless in the past year due to their gender identity [7].

One contributing factor to the greater rates of socioeconomic instability is the social stigma that results in many TGD individuals receiving little or no family support [8]. Many have also not disclosed their gender identity to their families due to fears of rejection [9]. As a result of this stigma, TGD people are more likely to lack strong support networks and experience adverse social conditions that harm their mental health. Discriminatory stresses have been found to contribute to psychological distress and worsen suicidal ideation in TGD people [10]. According to the 2015 US Transgender Survey, 39% of the respondents experienced serious psychological distress at the time of the survey and 40% of the respondents have attempted suicide, which is nearly nine times greater than the general US population [7]. Another survey in New Zealand comparing the rates of psychological distress between TGD and cisgender people (N = 905) found that 72% of the respondents had high or very high psychological distress symptoms, almost two standard deviations above the general population [11]. This social stigmatization and discrimination contribute to significant negative effects on the physical and mental health of the TGD population.

Even when accessing healthcare, TGD patients often face discrimination from healthcare providers. In a national sample of transgender males in the United States (N = 1,711), 41.8% of the participants reported harassment, physical assault, or denial of treatment from healthcare providers [12]. In another survey (N = 3,486), nearly a third of participants (30.8%) chose not to pursue healthcare due to fears of physical assault and harassment by healthcare providers [13]. A lack of provider cultural competency regarding transgender health issues also remains a major barrier to equitable healthcare. Concerns such as being unintentionally misgendered, misunderstanding of social stigma, and lack of knowledge often necessitate TGD patients to educate their providers [14]. Therefore, addressing cultural competency and hostility from providers is crucial to promote a more inclusive clinical environment for all patients.

Training and medical education

The inclusion of TGD-specific content across all ranges of medical education has been strongly recommended by the American College of Physicians, the Association of American Medical Colleges (AAMC), and other medical institutions to improve knowledge of TGD healthcare [15,16]. However, there is a paucity of curricula incorporating TGD-specific healthcare concerns across all levels of medical education. A survey of Liaison Committee on Medical Education-accredited faculty practices in the USA reported 52% having no lesbian, gay, bisexual, transgender, and queer/questioning (LGBTQ) training and only 16% having a comprehensive LGBTQ curriculum [17]. Another study of Australian and New Zealand medical education reported that 60% of schools had 0-5 hours of LGBTQ content in their preclinical curriculum [18].

This lack of TGD medical education has resulted in unfamiliarity and discomfort for practicing physicians to work with TGD patients. One survey of gynecologists (N = 141) revealed that 80% did not receive training and only a third were comfortable providing care for TGD patients [19]. Another survey of endocrinologists (N = 147) found that only 19% were confident in their ability to provide appropriate clinical care to transgender patients. Respondents also reported that only 4% received training on TGD care in medical school. However, 91% indicated a desire for additional training [20].

The issue extends to residency training as well. In a survey of family medicine, psychiatry, endocrinology, and urology residents (N = 319), 17% reported feeling competent enough to provide trans-specific care, and only 12% felt that their training adequately met the needs of the transgender patient population [21].

## Materials and methods

The framework approach utilizes a systematic procedure to analyze qualitative data via thematic analysis [22]. This method promotes an unobtrusive form of data collection, where participants are not directly interacted with. Anonymity complements the data collection procedure as data is authentic to their experiences and participants may feel less apprehensive to speak on sensitive topics [4]. Furthermore, adopting an inductive thematic framework enables standardized coding, labeling, and organization of data under broader themes and categories. This method emphasizes the flexible, iterative nature of the coding process, in which codes and themes constantly shift and reform throughout each stage.

The approach in practice involved analyzing relevant threads from the major social media forums r/asktransgender, r/trans, r/transgender, and TransPulse Forums across three major stages: data collection and management, descriptive accounts, and explanatory accounts. Inclusion criteria prioritized relevance, with threads being identified for pertinence concerning provider knowledge of transgender health, transgender health in medical education, and descriptions of clinical interactions. An initial search employed two strategies, with the first using relevant search terms on a generic search engine to identify the forums of interest and the second using search terms on the sub-forums’ internal search engines to locate admissible threads. Search terms were also combined (e.g., “medical education” and “doctor” or “healthcare training” and “experiences”) to improve search results. Furthermore, search results on the internal forum search engines were filtered by both “relevance” and “top” for the most popular relevant posts. Other inclusion criteria included recency, with the most recent thread posted in 2023 and the earliest in 2014, and high levels of engagement with other members of the community via comments and votes. Threads were excluded for an emphasis on non-related topics such as transitioning guidelines or metaphysical health, posts prior to 2014, and low community engagement.

The final dataset consisted of a total of 23 threads, with 21 from the social media forums r/asktransgender, r/trans, and r/transgender and two from the TGD support group TransPulse Forums. The most commented thread had 192 relevant responses, and the least commented thread had four. From the 23 threads, a total of 468 text-based posts were inductively coded using the framework approach. Text drawn from coded data for results was slightly edited without a change in tone or intent to preserve poster anonymity.

The first stage involved data collection and management, whereby coded data was assigned to initial themes and categories within a five-column coding matrix. Codes were assigned iteratively through each column of the matrix, moving through a transcription of the post’s text, a description of the text, in vivo codes (specific phrases taken from the post’s text), preliminary impressions, and initial code. Following this pattern ensures that the poster’s intent is accurately interpreted prior to developing initial codes and themes. The descriptive accounts stage then worked toward refining initial coded data by noting links between codes, identifying common trends, and holistically synthesizing information in a three-column coding index. Similar to the coding matrix, each column gradually informs the next in an ordered fashion. The index includes an initial code, initial categories, and an initial theme. As new codes developed through a continuous process of refinement, the characteristics of coded information emerged more clearly in relation to an overarching theme. Finally, the explanatory accounts stage drew conclusions from refined themes and codes, mapped connections between categories to explore causality, and fully interrogated theoretical data for novel insights. In this stage, final themes are established and connected to each coded text. Initial coded data was compared with the final themes to reflect on the original intent of posters and avoid misinterpretation during the refinement process.

## Results

Three central themes were generated from the analyzed media: desire for more knowledgeable providers, negative experiences in healthcare, and recommendations for transgender health education. In presenting these findings, the literature has been incorporated to support the following interpretations of each theme. The results are listed in Table 1.

**Table 1:** Coding of Final Themes

Themes and Subcategories		Description and/or Purpose	Total (468)	Total (100%)
Desire for more knowledgeable providers		Improve access to providers capable of managing transgender and gender-diverse health concerns in a respectful and competent manner	94	20%
Negative healthcare experiences and attitudes		Discrimination, stigma, and ill-prepared healthcare that contribute to inappropriate clinical experiences	267	57%
Recommendations for transgender health education	Curriculum reform in medical education	Appeal for the integration of transgender health content in medical education	59	13%
	More inclusive medical training culture	Develop a culture of openness in medical education and promote culturally sensitive care	17	4%
	Encourage health careers for TGD individuals	Improve transgender and gender-diverse representation in healthcare to reflect the patient population	31	6%

Desire for more knowledgeable providers

Approximately 20% of the transgender posters reflected a desire for more knowledgeable providers. Many commiserated on the lack of providers available in their region, and some even reported traveling across state lines for healthcare from a provider they trusted. While discrimination was an issue for some patients, a large proportion of posters wrote about providers not having the training or education necessary to manage their concerns. As one poster reports “*their* *(the providers) faces fell flat when they realized I was trans*.” From the perspective of many participants, this attitude did not stem from provider malice but was rather from posters feeling that their provider was unaware of how to manage a patient who was transgender. As a result, they felt less confident in their provider’s ability to deliver healthcare appropriate to their needs (“*Many (TGD patients) have to educate their physicians about trans identity and transgender health*”).

Other posters reflected on the trans broken arm syndrome, a phenomenon where a transgender person could enter a doctor’s office with a severe concern such as a broken arm and their provider attributes their health concerns to being transgender. This is considered a consequence of a lack of education and training among medical providers to manage the concerns of TGD patients independent of their gender identity (“*Flu, spider bite … everything written as caused by being trans”*).

Access to high-quality, sensitive care was limited for many forum participants. Comments would reflect a strong desire for providers aware of gender-related treatment and resources, with some avoiding healthcare altogether if they felt that their provider was ill-equipped to handle transgender health in a competent manner (“*I’d rather use my doctor that also does my (hormonal replacement therapy), if only because I am certain the other places are completely incompetent*”).

Specific nuances and insights into specialized services were not expected from every provider, but posters felt a grasp of basic gender information and resources was important for providers to have. Furthermore, references to culturally responsive healthcare professionals were perceived as helpful and supportive. Examples of resources include transgender health organizations, mental health providers, transition support services, and educational media.

Negative healthcare experiences and attitudes

Posters often characterized the current state of transgender healthcare, reporting discrimination, stigma, and a lack of knowledgeable providers. They faced quotidian violence amplified over the course of their healthcare experiences, and over 57% of the data reported such clinical interactions (“​​​​​​*So far, my experience with healthcare has been horrible*”). For example, misgendering frequently contributed to gender dysphoria (“*The entire thing misgendered me*”), patients had to make health decisions due to a lack of provider knowledge, and stereotyping was common.

Some posters expressed doubt over the quality of transgender medicine research and its clinical applications, with a large portion of skepticism informed by short-term assessments and one-time interventions developed for medical training. Over one discussion, this suspicion fell onto medical sciences as a whole as some advocated turning toward alternative medicine instead.

Despite the mistrust, there is a clear trend among the many negative healthcare experiences reported by TGD patients where posters do not narrate their providers as explicitly transphobic but rather as healthcare professionals who lack the experience, knowledge, and comfort to adequately manage transgender health concerns in a well-informed manner.

Recommendations for transgender health education

Some forum participants challenged traditional medical training and argued in favor of broader reforms that incorporated transgender health concerns. Three smaller subcategories emerged, with the first calling for curricular change in medical schools and residency training that was inclusive of transgender health (13%). In particular, posters emphasized culturally responsive care that approaches TGD patients with humility, intersectional approaches that account for the interconnected nature of health disparities including class and race, and integrating transgender patients into the training process (“*It would be a big step forward to get it (transgender medicine)* *standardized … in medical curricula*”).

The second subcategory initiated a demand for a learning environment that challenges gender as a binary and instead considers a comprehensive approach where the complexity of gender can be reflected on. Posters consider how trans-specific content on gender variation should be embedded throughout the curriculum. Otherwise, posters indicate that separating transgender identities into categories distinct from cisgender identities reinforces the pathologization of transgender patients as an “unhealthy other.” Although this subcategory includes the lowest proportion of data at 4%, other recommendation categories frequently included coded data that would complement a cultural change in clinical training (“*(Medical students) have rigid schemas about gender. Break those ideas down. Teach them to *…​​​​​​​ *question without judgment*”).

The final subcategory highlighted the inclusion of more TGD providers in healthcare (6%). Posters would cite the importance of including TGD providers to promote healthcare access as “*seeing someone who is like me in the room makes a difference.*” Clinicians who reflect the population they work with tend to improve the comfort and attitude of patients toward their healthcare experience [23]. Many participants also spoke on personal aspirations in the field and on reforming medical education from within the institution. They advocated for careers accessible to TGD people and called for healthcare workers to accurately represent the proportion of TGD patients.

## Discussion

Many of the recommendations, desires, and provider preferences argued by posters to better prepare healthcare professionals for a diverse patient population were supported by the literature on transgender medical education. Broad themes ran parallel to reviews of TGD health training and reflected the crucial need for the institutionalization of TGD content throughout the medical training process [24]. More specifically, quotes regarding curricular changes to medical education echoed common interventions to improve training. Authors highlighted adopting TGD health electives in medical school curricula, including student-delivered presentations, elective rotations, interactive online modules, and standardized patient panels that included TGD patients [24,25].

A common theme among these strategies was to emphasize active learning modules that positioned students in direct contact with TGD patients through role-play patient encounters and clinical scenarios, reflecting poster desires for additional research that included transgender patients. One study reports on a half-day transgender health intervention, including case presentations, live patient interviews, and patient question and answer (Q&A) sessions. This model included an examination that incorporates appropriate hormone regiments and anatomy-based cancer screening protocols. Reflecting on forum poster desires for providers more familiar with transgender health concerns, the results revealed a significant improvement in students’ comfort with and knowledge of transgender healthcare [26].

Another pilot initiative at the Boston University School of Medicine rotated students through clinics providing care for transgender patients and reported similar improvement in comfort in treating TGD patients [27]. Longitudinal experiences in TGD healthcare have also been developed. At the Yale University School of Medicine, students and faculty implemented an integrated curricular sequence that complemented the preexisting curriculum with active learning modules on TGD patients [28]. In accordance with the desires and recommendations of TGD patients in online media, the prevailing conclusions among TGD health education interventions identify significant improvements in trainees’ comfort with, knowledge of, and willingness to provide care for transgender people [24-28].

Findings from this study are also consistent with another highlighted strategy to include transgender health consultants as facilitators and improve access to provider careers. Exemplary quotes drawing on the lived experiences of TGD people can enable more informed medical decisions and ensure authenticity in clinical scenarios. In line with the literature base, engaging with transgender community members has been found to yield invaluable insight and support sustainable curriculum delivery [23].

Even participant concerns regarding the short-term nature of current research reflect the limitations of transgender health interventions. One study reported on a one-time intervention that produced immediate increases in provider comfort with TGD patients, but an extended follow-up found provider professionalism returned to baseline [29]. This report, along with poster apprehension with TGD medical research, highlights the need for longitudinal efforts that integrate TGD health concerns across medical education and professional training as opposed to one-time events. Of those that implemented such longitudinal curricular reforms, the changes were implemented recently and lack long-term measures of effectiveness in clinical practice [28,29]. However, the trend toward the institutionalization of TGD health education has been established as an important mechanism to shift the culture of medical education and highlight gender variation as a normal component of human diversity [30].

This study is limited by the analysis of online forum text, much of which could be highly exaggerated, not drawn from personal experience, or deceptive. Since it was not possible to identify who posted each comment in the forums, it is not possible to test the veracity of many claims. Furthermore, it is not possible to gather demographic data for an expansive perspective of transgender health experiences. For example, TGD people in online settings can be skewed to wealthier patients who may have better access to health resources or to other groups that frequent online discourse more often than others. Other factors such as location, age, and ethnic background were also not able to be collected. However, online data can be a potent source of naturalistic research and a valuable starting point to address patient concerns.

## Conclusions

Transgender and gender-diverse patients face numerous systemic barriers in clinical settings, including discrimination, stigma, and a lack of knowledgeable providers. Furthermore, a large proportion of providers are not fully prepared to address the unique healthcare concerns of this patient population. Through an analysis of online forums concerning transgender healthcare, this paper argues that medical education must complement preexisting medical training curricula to equip providers with the knowledge, tools, and experience to manage the concerns of gender-diverse patients. In line with this study’s examination of participants in online media via an inductive thematic analysis, building a foundation of providers proficient in transgender healthcare will require longitudinal curricular reforms, community engagement, and a shift toward a more inclusive medical culture.
